# Merging and scoring molecular interactions utilising existing community standards: tools, use-cases and a case study

**DOI:** 10.1093/database/bau131

**Published:** 2015-02-03

**Authors:** J. M. Villaveces, R. C. Jiménez, P. Porras, N. del-Toro, M. Duesbury, M. Dumousseau, S. Orchard, H. Choi, P. Ping, N. C. Zong, M. Askenazi, B. H. Habermann, Henning Hermjakob

**Affiliations:** ^1^Max Planck Institute of Biochemistry, Am Klopferspitz 18, 82152 Matinsried, Germany, ^2^European Molecular Biology Laboratory, European Bioinformatics Institute (EMBL-EBI), Wellcome Trust Genome Campus, Hinxton, Cambridge CB10 1SD, UK, ^3^Department of Physiology and ^4^Department of Medicine, Division of Cardiology, David Geffen School of Medicine at UCLA, 675 Charles E. Young Drive, MRL Building, Suite 1609, Los Angeles, California 90095, USA and ^5^Biomedical Hosting LLC, Arlington, Massachusetts 02474, USA

## Abstract

The evidence that two molecules interact in a living cell is often inferred from multiple different experiments. Experimental data is captured in multiple repositories, but there is no simple way to assess the evidence of an interaction occurring in a cellular environment. Merging and scoring of data are commonly required operations after querying for the details of specific molecular interactions, to remove redundancy and assess the strength of accompanying experimental evidence. We have developed both a merging algorithm and a scoring system for molecular interactions based on the proteomics standard initiative–molecular interaction standards. In this manuscript, we introduce these two algorithms and provide community access to the tool suite, describe examples of how these tools are useful to selectively present molecular interaction data and demonstrate a case where the algorithms were successfully used to identify a systematic error in an existing dataset.

## Introduction

To understand the behaviour of molecules such as proteins in the living cell, an understanding of their interactions with other molecules is critical. Protein interaction data are generated by many different methodologies in low or high throughput. The results from interaction studies are scattered across a broad spectrum of biological publications. This information is collected by the many interaction databases in existence today (1, 2). In isolation, each piece of experimental data can only contribute to the understanding of one specific biological process, but the combination of all interaction data gives researchers an overall picture of the relationships between molecules in a cell, a tissue or an organism. Consolidation of this data is thus essential for the research community to give the most complete data representation possible.

Dedicated teams of curators collect molecular interaction data from literature and accurately represent this information in a structured database. The type and amount of information captured by different curation groups varies in different resources. Rapid curation records only minimal information about either the experiment or participating molecules, MIMIx-level curation (3) gathers experimental detail, but not additional information about the participating molecules provided by the detailed IMEx-level curation (4), which describes all possible details the authors give concerning a specific experiment and its molecular components. It is, however, particularly important that all experimental details under which each interaction was observed are recorded. The field currently lacks a single methodology, which can unambiguously identify a molecular interaction as being physiologically relevant in the intact, living cell. All current methods for detecting protein– protein and other molecular interactions are capable of generating false-positive data. However, by combining observations made using different experimental methodologies, it is possible to increase the confidence with which the researcher can regard a particular interaction. If a specific interaction has been confirmed by multiple observations and/or experimental methodologies, more confidence can be assigned to it. Despite over 10 years of work, no database, nor indeed a compilation of all available scientific data generated to date, can claim to fully describe the interactomes of even well-studied model organisms such as *Saccharomyces cerevisiae* or *Homo sapiens*. Thus, many resources attempt to improve coverage by inferring through computational approaches (e.g. phylogenetic profiling, association methods, inference of interactions from homologous structures) those interactions that are not reported in the literature. Though predictive data would not be expected to be as trustworthy as experimental data, both are important to assess the overall evidence for an interaction.

Integration and comparison of data is essential to increase the coverage of an entire interactome, but also to increase confidence in a single interaction within an interactome. Starting from 2002, the Human Proteome Organisation Proteomics Standards Initiative (HUPO-PSI) has made an effort to develop molecular interaction data standards, data interchange formats and controlled vocabularies with which to implement these standards in a consistent manner (5, 6). The adoption of Proteomics standard Initiative–Molecular Interaction (PSI–MI) standards by data providers and software tools has played an important role in facilitating data integration. It is now easy to query interactions from diverse and distributed interaction resources and group evidences relating to the same interaction.

The community has, however, not agreed yet on a generally accepted common scoring system for molecular interactions (7)]. A set of different confidence measures for molecular interactions exist. Many of these are specific to particular experimental methodologies, for example, yeast 2-hybrid (8) or affinity purification coupled with tandem mass spectrometry (9). Others use heuristic integration of annotation evidences with third-party data such as the results of text-mining or Gene Ontology annotation of the interacting protein pairs (10, 11). Scoring the interactions according to the known topology of the network, the ‘wiring diagram’ of the cell, is the basis of another popular set of methodologies (12, 13). However, the field is still lacking a simple implementation of a confidence scoring methodology, which works over any standards compliant dataset and can readily be used by bench scientist to assess the quality of their own data prior to publication using code made publicly available to enable this. With the objective of providing reusable tools for integrating and scoring molecular interactions evidences, we present MImerge and MIscore. The MImerge service groups and merges evidences for the same interaction. MIscore provides a customizable scoring system reliant on the annotation of experimental, predicted or inferred data from which each interacting binary pair was generated using the PSI–MI standards and format.

## Methods

### MImerge

**Figure 1.**
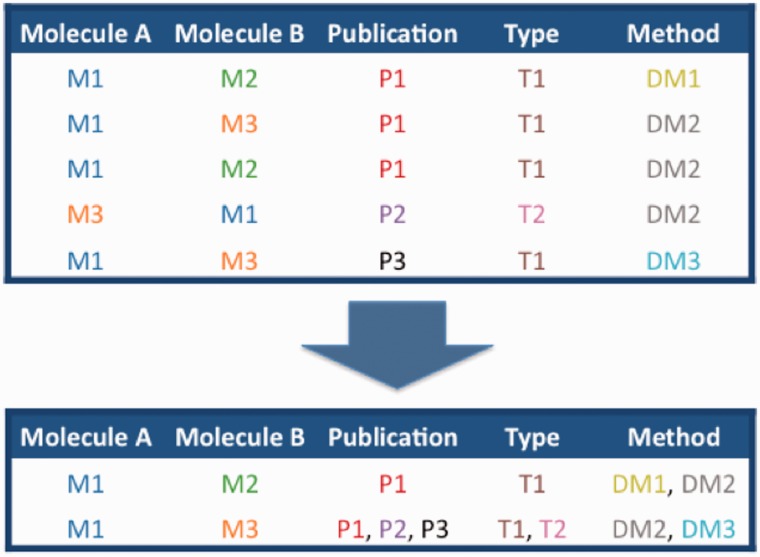
Schematic of the merging of interactions between molecules M1–M3, described in publication P1–3 by interaction detection methods D1–3 and with interaction types T1 and T2.

MImerge recognizes groups of evidences of the same interaction, merging redundant annotations and identifying novel information (Figure 1). Merging is performed by matching interacting molecule pairs using a predefined set of database identifiers and cross references. The algorithm matches interactor molecules based on standard identifiers such as UniProtKB (14), RefSeq (15), ROGID (16), or ChEBI (17) accession numbers.

#### Input

The primary input of MImerge is a binaryInteraction java object defined by the PSI MITAB java implementation [http://code.google.com/p/psimi/]. Alternatively, the method accepts interactions in any of the versions of the PSI MITAB formats (5, 6). To facilitate data retrieval, MImerge can connect, query and fetch interaction data from any of the Proteomics Standard Initiative Common QUery InterfaCe (PSICQUIC) (7) services available in the PSICQUIC registry.

#### Output

MImerge provides three outputs:

- a list of interactions
- a list of interactors
- a list of interactor synonyms

Each interaction is the result of merging all the experimental evidences indicating that a specific pair of molecules interacts. The primary output is an object containing all the new information provided by the original evidences. The ‘interaction objects field’ also retains the original relationship information in fields such as publication, interaction type and detection method allowing, for example, the separate scoring of all the individual pieces of evidence for a particular binary interaction, which can then be resolved into a single cumulative score. This primary object can be easily exported into a merged PSI–MI MITAB25 format. Thus the algorithm produces a list of interactors with both references to the interaction results and a list of synonyms found for each interactor.

#### Mapping

The PSI–MI formats provide three different fields in which information used to identify an interactor can be stored: (1) the unique identifier, (2) alternative identifiers and (3) the aliases. MImerge accesses these three fields to identify cross-references, which could potentially identify molecules with different identifiers but describing the same entity. More details including examples of how to use MImerge are available in http://code.google.com/p/micluster/

### MIscore

MIscore is a customizable, heuristic scoring system that does not rely on a comparison with third-party data but rather on the available annotation evidences associated with an interaction. It is capable of scoring any type of interaction evidence (experimental, inferred, predicted) adhering to the MIMIx guidelines and being described using the PSI–MI controlled vocabulary (CV) (5). The method is agnostic to the type of interactor, working equally well for protein–protein interactions, protein– nucleic acid, drug-target or any combination of molecular interactions. The PSI–MI data formats include a field in which molecule type should be clearly defined, according to an agreed set of CV terms, so the user may pre-filter out molecule types which they do not wish to merge. Similarly, the PSI–MI file uses CV terms to describe the experimental, predicted or inferred evidence used to identify a specific interaction. If the users only wish, for example, to work with experimental data, they can filter the file first, remove all predicted data and then run MIscore. Detailed annotations will also score more highly than less detailed ones. For example, use of a top-level term such as ‘experimental interaction evidence’ will score less well than a more detailed annotation of the methodology, such as ‘X-ray crystallography’. The scoring system takes three factors into account:

1. How the interaction was observed, predicted or inferred (interaction detection method; MI:0001)
2. The type of interaction. Direct interaction, physical association, co-localization and so forth. (interaction type; MI:0190)
3. The number of publications reporting a specific interaction

MIscore provides a score that represents the degree of confidence in the existence of a particular interaction by assessing the annotation of that specific interaction in a standards-compliant dataset. The score given to an interaction will increase as the number of experimental evidences supporting that interaction increases. Experimental evidences contribute more highly to the final score than evidences derived by predictive algorithms or literature text-mining methods. Combinations of evidences, such as low scoring experimental interactions (e.g. co-localizations) supported by non-experimental evidence provide a higher degree of confidence than either would in isolation. In the versions of MIscore implemented by the IntAct database and for the filtering of data for export from IntAct to UniProtKB, the values have been selected to reflect the ethos of these databases, with a strong emphasis on there being experimental evidence for the existence of a physical interaction. Full details of the scores used as available on the IntAct ‘FAQ—Frequently Asked Questions’ section. Databases such as BioGRID (18), which captures genetic evidences for an interaction, may prefer to use different weighting when implementing this scoring system, and the algorithm has been specifically designed to enable this.

In Table 1, the evidence for AKT interacting protein (AKTIP) binding to hook microtubule-tethering protein (HOOK2) in various databases has been merged and scored. IntAct provides fewer pieces of evidence than STRING (11) but scores higher because it offers detailed experimental evidence of a direct interaction. A meta-database such as Mentha (19), that integrates experimental evidences from different sources, gives an even higher score (0.76 in the case of this specific protein pair). If we look for experimental evidences in all the PSICQUIC services, we find 12 evidences from five different databases resulting in a high confidence score of 0.81. Thus, merging the predictive and experimental evidences increases the confidence score for this interaction.

**Table 1.** Merging and scoring evidences of the interaction between AKTIP_HUMAN and HOOK2_HUMAN

PSICQUIC service	Interaction evidences	Publications	Interaction types	Detection methods	MIscore
STRING	3	1*	–	Experimental interaction detection Inferred by curator Predictive text mining	0.20
VirHostNet	1	1	Physical association	Two hybrid	0.37
Spike	1	1	Direct interaction	Coimmunoprecipitation	0.44
IntAct	2	2	Physical association	Two hybrid pooling approach Two hybrid fragment pooling approach	0.35
APID	1	1	Association	Two hybrid pooling approach	0.31
Menthe	7	3	Physical association Direct interaction	Affinity chromatography technology Two hybrid Two hybrid pooling approach Two hybrid fragment pooling approach	0.76
Spike IntAct VirHostNet	4	2	Direct interaction Physical association	Two hybrid Coimmunoprecipitation Two hybrid pooling approach Two hybrid fragment pooling approach	0.68
APID mentha Spike IntAct VirHostNet	12	3	Direct interaction Physical association Association	Two hybrid Coimmunoprecipitation Two hybrid pooling approach Two hybrid fragment pooling approach Affinity chromatography technology	0.81
Spike IntAct VirHostNet APID mentha STRING	15	3	Direct interaction Physical association Association –	Two hybrid Experimental interaction detection Inferred by curator Predictive text mining Coimmunoprecipitation Affinity chromatography technology Two hybrid pooling approach Two hybrid fragment pooling approach	0.81

MIQL query “identifier:(Q9H8T0) AND identifier:(Q96ED9)”. *Predicted data from STRING does not have any publications assigned, so publication number here is attributed only for experimentally derived data, which is imported from other databases.

### Score calculation

**Figure 2.**
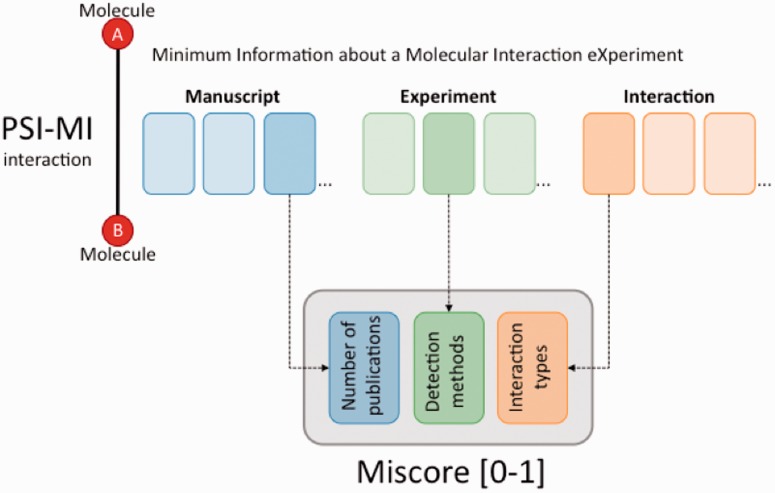
The MIscore normalized score calculates a composite score for an interaction based on the number of publications reporting the interaction, the reported interaction detection methods and interaction types.

By default MIscore presents a normalized score (*S*_MI_) between 0 and 1 reflecting the reliability of its combined experimental evidence. This score is calculated from the weighted sum of the three different sub-scores listed above: number of publications (*p*), experimental detection methods (*m*) and interaction types (*t*) found for the interaction (Figure 2). The importance of each variable in the main equation can be adjusted using a weight factor. Each of these sub-scores is also represented by a score between 0 and 1.

SMI=Kp×Sp(n)+Km×Sm(cv)+Kt×St(cv)Kp+Km+KtK[p,m,t]≡Weight factor  ||  K∈[0−1]S[p,m,t]≡Scores  ||  S ∈[0−1]

### Publication score

The publication score takes into account the number of different publications supporting an interaction.

Sp≡Publication Score  ||  Sp ∈[0−1]Sp=log(b+1)(n+1)

*n *≡ Number of publications reporting the interaction $\left|\right|\;S_{p\;}\equiv n\;\in N\;[0,1,2,3\;...]$.

*b* ≡ Number of publications with maximum score; default: b = 7

### Method score

The method score takes into account the diversity of interaction detection methods reported for an interaction.

Sm≡Method Score  ||  Sm ∈[0−1] Sm(cvi)=log(b+1)(a+1)a=∑(scvi×ni)b=a+∑(Max(Gscvi))

 

*scv* is a normalized score between 0 and 1 associated to an interaction detection method term, as defined by the MI ontology. An MI detection method ontology term without an assigned score inherits the score from the nearest parent. *Gscv* represents a category of scores normally grouping scores with a common parent. *n* is the number of times an ontology term is reported. The *scv* score values are customizable; however, detection method ontology terms are assigned with a default score based on the assessment of the HUPO PSI–MI consortium:

scv1 = 1.00 || cv1 = MI:0013 | biophysical

scv2 = 0.66 || cv2 = MI:0090 | protein complementation assay

scv3 = 0.10 || cv3 = MI:0254 | genetic interference

scv4 = 0.10 || cv4 = MI:0255 | post transcriptional interference

scv5 = 1.00 || cv5 = MI:0401 | biochemical

scv6 = 0.33 || cv6 = MI:0428 | imaging technique

scv7 = 0.05 || cv7 = unknown | unknown

Gscv1 = scv1 | Gscv2 = scv2 | Gscv3 = scv3 | Gscv4 = scv4|Gscv5 = scv5 | Gscv6 = scv6

### Type score

The interaction type score takes into account the diversity of interaction types reported for an interaction.

*S_t_* ≡ Type Score | | S_t∈_ [0−1]

*S_t_*(*cv_i_*) = log_(*b* + 1)_(*a* + 1)

*a* = ∑(*scv_i_* × *n_i_*)

*b* = *a* + ∑(*Max*(*Gscv_i_*))

As in the method score, *scv* is a normalized score between 0 and 1, in this case associated to an interaction type CV term. An MI-type ontology term without an assigned score inherits the score from the nearest parent. Interaction-type scores are also customizable but by default they have assigned a heuristic score based on the assessment of the HUPO PSI–MI consortium:

scv1 = 0.10 || cv1 = MI:0208 | genetic interaction

scv2 = 0.33 || cv2 = MI:0403 | colocalization

scv3 = 0.33 || cv3 = MI:0914 | association

scv4 = 0.66 || cv4 = MI:0915 | physical association

scv5 = 1.00 || cv5 = MI:0407 | direct interaction

scv6 = 0.05 || cv6 = unknown | unknown

Gscv1 = scv1 | Gscv2 = scv2 | Gscv3 = scv3, scv4, scv5

More details including examples of how to use MIscore are available at https://code.google.com/p/miscore/.

## Results

### Tools

A number of services have been built based on MIscore and MImerge, which allow users, with or without technical skills to merge and score interaction evidences. All these services are open source and available under the ‘GNU GPL v3’ license.

#### 1. Java APIs

Java APIs are available for MIscore and MImerge (https://code.google.com/p/miscore/ and https://code.google.com/p/micluster/, respectively). MImerge includes MIscore as a dependency, providing the option of merging interactions and scoring groups of evidences. An API has also been implemented to calculate the score distribution of a collection of interactions from a database.

#### 2. Web services

To facilitate programmatic access, a REST web service based on a MImerge API is publicly available. The service permits users to merge and score interactions from PSICQUIC services using the PSI–MI query language (MIQL) or alternatively, from a PSI–MITAB file. Additionally, MIscore is available as a PSISCORE web service providing evidence scores based on data from PSICQUIC services (7).

The service provides three different methods, (i) ‘cluster’ that sends a request to the server to start a merging job and returns a job id; (ii) ‘status’ that returns the status of a particular merging job and (iii) ‘download’ that returns a PSI–MITAB file containing the processed interactions. To prevent abuse, the service stops automatically if the merging takes more than a day or if the input file is >5 MB.

#### 3. Web interface

To enable human access to the web service and as an example of a use case for the web service, a web interface has been built (http://dachstein.biochem.mpg.de:8080/mimergeclient/). The interface inherits all the functionality available in the web service for MImerge and MIscore.

### MIscore

To evaluate the performance of MIscore, we created a positive and a negative dataset. Interactions from Mentha were downloaded and the datasets were built according to the following criteria:

*Positive dataset selection*: the interactions have been reported (i) by three or more detection methods and (ii) in humans. At the time of writing, 12 778 unique interactions met the specified standards out of which a random subset of 500 was selected, evidences for the selected interactions were collected, merged and scored.
*Negative dataset selection*: the interactions have been reported (i) by the Negatome Database (43) and (ii) in humans. At the time of writing, 397 unique interactions met the specified standards, evidences for those interactions were collected, merged and scored.

**Figure 3.**
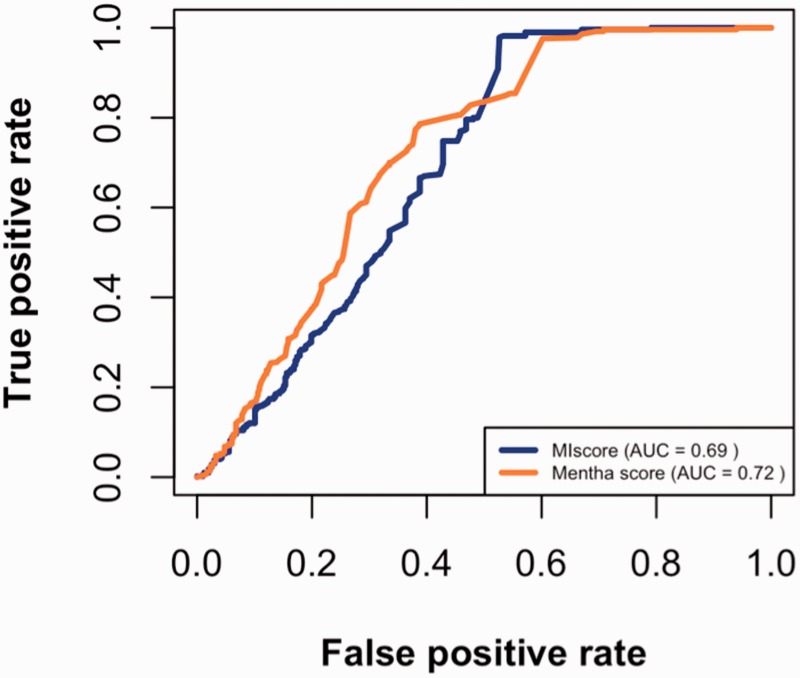
MIscore and Mentha true-positive rates vs. the false-positive rates for different score cutoffs.

Using the datasets described above, true positive and false positive rates were calculated for different cutoffs and then plotted (Figure 3). The figure suggests that MIscore and Mentha perform similarly since ROC curves have comparable area under the curve (AUC). The Mentha ROC curve rises steeply, which is consistent with higher precision. However, the MIscore ROC recovers at the end.

The maximal Matthews correlation coefficient (MCC) was calculated to find the cutoff point for optimal score predictions. As seen in Table 2, the optimal cutoff value for MIscore is 0.485 (which is close to the heuristic cutoff of 0.45 proposed by IntAct) while Mentha score optimal cutoff value is 0.343.

**Table 2.** Performance measures used to evaluate MIscore and Mentha scores

Score	Accuracy	Precision	Recall	MCC	Cutoff
**MIscore**	0.755	0.701	0.978	0.541	0.485
**Mentha**	0.673	0.660	0.854	0.474	0.343

Finally, the precision, accuracy and recall of both scoring methods were calculated for the optimal cutoffs (Table 2). MIscore precision, accuracy and recall values are higher than those of Mentha, meaning that (at that point) MIscore classifies positives and negatives better than Mentha.

### MImerge

MImerge was used to merge interactions from IntAct, BioGRID, MINT and DIP (20). At the moment of writing, the databases provided a total of 1 291 743 interactions, which were reduced to 865 642 after the merging process, implying that almost 33% of the interactions reported are redundant.

A closer inspection of the resulting data reveals BioGRID appeared to have no interactions in common with the other databases. That is not surprising since BioGRID annotates interactors using entrez gene ids and does not provide UniProtKB accessions in their MITAB download (as do the other databases) making it impossible for MImerge to find common interactions between BioGRID and the rest of the selected data providers.

**Figure 4.**
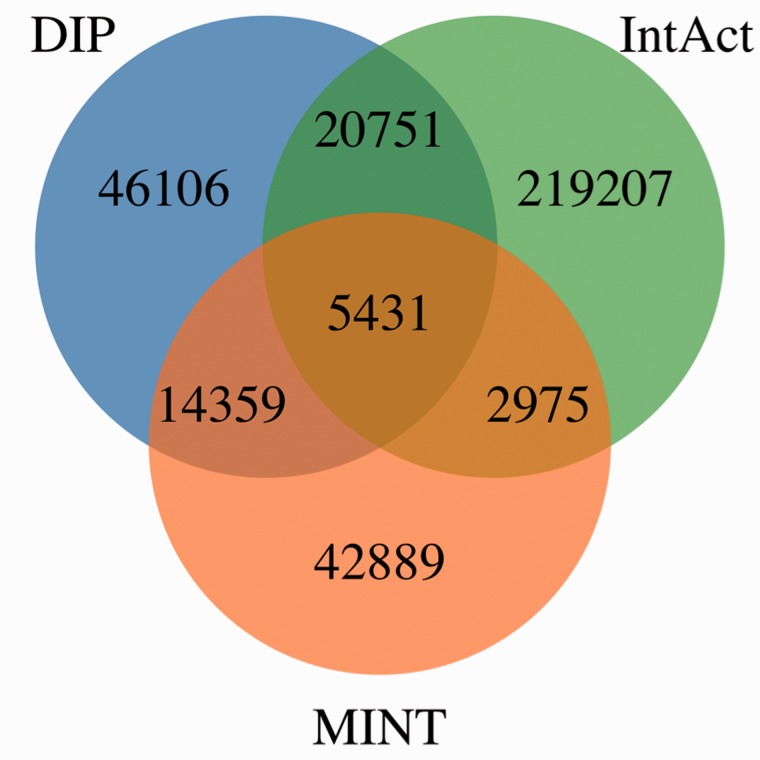
MImerge results for DIP, IntAct and MINT. Only 1.54% of the interactions are shared between the three databases, 10.86% are shared between two databases and 87.6% are not shared at all.

Figure 4 shows MImerge results for DIP, IntAct and MINT. Only 1.54% of the interactions are shared between the three databases, 10.86% are shared between two databases and 87.6% are not shared at all. The low redundancy values observed in the aforementioned databases are explained by the aim of curating different parts of the literature to increase coverage of the annotated interactome, an IMEx curation policy.

### Score distribution analysis across molecular interaction data providers

**Figure 5.**
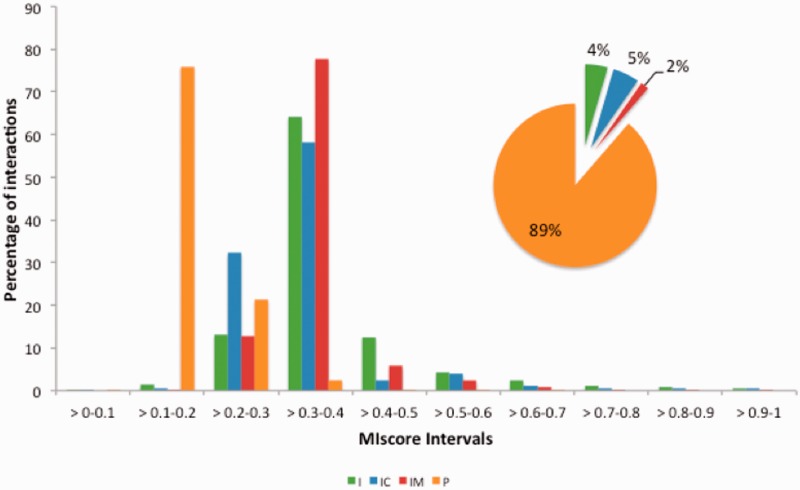
MIscore distribution proportion for the molecular interaction databases in Table 3. Databases have been grouped in four categories based on the type of evidences provided: imported (I), internally curated (IC), IMEX curated (IM) and predicted(p).

MImerge and MIscore were used to calculate the score distribution across several molecular interaction databases (Table 3). Databases have been grouped into four categories based on the type of evidences served: (i) internally curated (IC), (ii) IMEX curated (IM), (iii) predicted (P) and (iv) imported (I) (Figure 5). Not surprisingly, IMEX curated databases tend to have a proportionally higher score distribution since IMEX defines a common curation strategy that aims to provide a high standard dataset, whereas databases serving predicted evidences tend to have a lower score due to lack of additional support to prove an interaction.

**Table 3.** Merging and scoring evidences of interaction databases in PSICQUC

PSICQUIC service	Category	Interactions in interval											
		>0–0.1	>0.1–0.2	>0.2–0.3	>0.3–0.4	>0.4–0.5	>0.5–0.6	>0.6–0.7	>0.7–0.8	>0.8–0.9	>0.9–1	Total	Redundancy %
APID (21)	I	0	0	26 821	259 405	21 110	5480	4948	2406	1781	628	322 579	22,48
iRefIndex (16)	I	277	15 703	67 538	163 034	39 933	16 547	7566	3761	2659	1817	318 835	51,58
Mentha (19)	I	0	0	49 645	274 628	76 109	22 851	11 860	5074	2751	1847	444 765	37,31
BIND (22)	IC	0	3590	85 153	20 947	4891	1481	520	196	87	24	116 889	39,41
BindingDB (23)	IC	0	0	957	67 369	267	4440	1052	477	429	502	75 493	26,10
BioGrid (18)	IC	0	0	271 005	173 966	21 372	19 477	8107	5194	4805	2679	506 605	31,61
ChEMBL (24)	IC	0	0	30 020	437 441	1957	22 259	4005	1538	1205	1136	499 561	20,52
HPIDb (25)	IC	0	0	15	712	109	52	15	6	1	0	910	37,20
InnateDB (26)	IC	0	1	803	12 234	980	926	350	170	126	62	15 652	36,54
Spike (7)	IC	0	1	18 923	15 399	610	145	0	0	0	0	35 078	3,23
TopFind (27)	IC	1437	3334	178	5	2	0	0	0	0	0	4956	48,06
VirHostNet (22)	IC	0	0	707	8629	953	355	129	56	29	14	10 872	21,26
Reactome (28)	IC	0	0	0	141 996	0	0	0	0	0	0	141 996	0,00
bhf-ucl (29)	IM	0	0	16	278	60	31	7	0	0	0	392	45,02
DIP (20)	IM	0	0	42 864	34 785	6817	1496	426	224	67	2	86 681	19,46
I2D-IMEx (29, 30)	IM	0	0	61	308	105	52	3	0	0	0	529	52,43
InnateDB-IMEx (26, 29)	IM	0	0	14	274	56	24	3	0	0	0	371	45,44
IntAct (29)	IM	0	8	4946	220 853	11 302	4713	1957	492	147	29	244 447	20,67
MatrixDB (31)	IM	0	0	326	129	64	22	4	2	0	0	547	35,27
MBInfo (32)	IM	0	0	33	272	58	31	9	0	0	0	403	36,83
MINT (10, 29)	IM	0	0	3620	52 217	3739	3010	936	349	92	19	63 982	46,87
MolCon (29)	IM	0	0	12	230	52	6	1	0	0	0	301	39,19
MPIDB (33)	IM	0	0	93	723	160	70	19	4	0	0	1069	39,23
UniProt (14, 29)	IM	0	0	247	4316	1064	552	180	32	14	0	6405	45,46
BAR (7)	P	29	6765	74 013	23 442	23	0	0	0	0	0	104 272	0,53
Interoporc (34)	P	0	0	208 558	0	0	0	0	0	0	0	208 558	0,00
Reactome-FIs (28)	P	0	0	209 988	0	0	0	0	0	0	0	209 988	0,00
STRING (35)	P	0	16 335 859	4 110 825	373 086	31 888	2353	64	1	0	0	20 854 076	19,93
**Total**		**1743**	**16 365 261**	**5 207 381**	**2 286 678**	**223 681**	**106 373**	**42 161**	**19 982**	**14 193**	**8759**	**24 276 212**	

Databases have been grouped in four categories based on the type of evidences provided: imported (I), internally curated (IC), IMEX curated (IM), and predicted (P). The scoring has been performed with values reflecting IntAct ethos.

**Figure 6.**
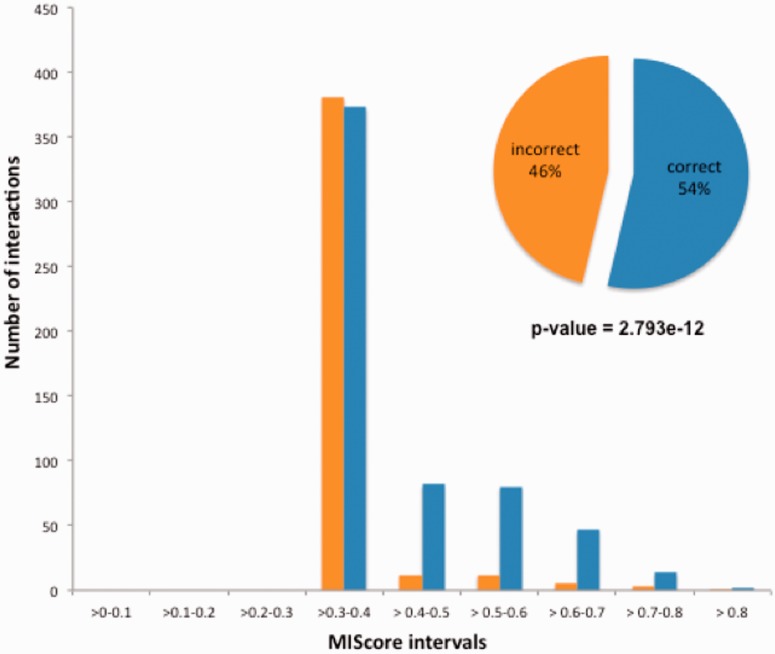
Distribution of IntAct MIscores for the pairwise interactions reported in Ref. 23. A clear and statistically significant difference in score distribution is evident between the 54% of the interactions which were correctly reported and the 46% which were effectively randomized. A Mood test for comparison of non-normally distributed samples was used to compare both groups.

Also, it was observed that databases that import predictions (in particular Mentha) show a score distribution increase, shifting scores to higher values, when compared with the databases they integrate. The score increases when merging evidences was further explored by merging and scoring evidences of the interaction between AKTIP_HUMAN and HOOK2_HUMAN (Table 1). MIscore does not provide an interaction quality score per se but rather a measure of how well annotated an interaction is. Therefore, it would be expected that the combination of evidences from different data sources contribute to increase the score. However, the observed increase is not as great as one might expect. The slight increase is due to the high number of redundant interactions, which are repeated by secondary databases and which do not add value to the score. Redundancy in molecular interaction databases can be high (29) largely caused by those databases that do not collect novel curation or predictions but rather import and present data from other interaction databases. MImerge removes such redundancies by merging interactions from secondary databases such as iRefIndex (16).

### Services using MImerge and MIscore to selectively display or import molecular interactions

MImerge and MIscore services are currently being used by several applications to filter, sort and select molecular interactions.

#### UniProt

An extended version of the MIscore is being used by UniProtKB (12), Gene Ontology annotation project (36) and NeXtProt (37). Those resources calculate scores of interactions from different IMEx databases (4) to selectively import interactions above a defined score threshold. Additional rules ensure these are true binary interaction rather than complex components, which frequently co-purify and thus score highly as interacting molecules.

#### IntAct

The IntAct database (32) and its web interface use MIscore to score molecular interactions. By default, the IntAct web interface displays interactions sorted according to the score provided by MIscore, with the most highly scoring binary pairs displayed first. When filtering data for subsequent reanalysis, the IntAct database regards data with a score of >0.6 as high-confidence and 0.45–0.6 as medium confidence but users are free to use their own cutoffs when using the Search tool to filter the data as they see fit.

#### EMBL-EBI search

The EMBL-EBI search (38) uses MImerge and MIscore to provide non-redundant summary information about molecular interactions, selecting specifically IntAct interactions with a high score.

#### PSICQUIC

MIscore scores are also available in several PSICQUIC services (UniProt, IntAct, MINT, ChEMBL (24), I2D-IMEx (30), InnateDB-IMEx (26), MBInfo (http://www.mechanobio.info), MolCon (http://www.molecularconnections.com) and UniProt). It is possible to query all these services by score using MIQL.

#### COPaKB

The Cardiac Organellar Protein Atlas Knowledgebase (39) presents interactome views for each proteome module. The interactomes are built using MImerge to integrate protein interaction evidences from IMEx resources. It also makes use of the weight of MIscore scores to create the interactome layout. [http://www.heartproteome.org/copa/Modules.aspx]

#### Cytoscape

Cytoscape (40) has added the option to merge and score PSICQUIC molecular interactions using MImerge and MIscore. This option is part of the core implementation in version 3.1 as ‘intact-MIscore’, a column that results from using the option ‘Automatic Network Merge (Experimental)’ in the import tool.

### A Case Study for Literature-Based Protein Interaction Curation

Literature curation provides useful reference sets for further data analysis, prediction and validation. A confidence score such as MIscore can play an important role in facilitating such tasks. As an example, we present an actual case of how MIscore was used to analyse a submission error in an experimental dataset of high-throughput protein interactions.

The IntAct database accepted in 2008 a submission request to curate a high-throughput experimental dataset of ∼700 interactions, which were subsequently published (41). After publication the authors discovered that one-third of the reported interactions were effectively randomized due to a data management error. This problem was reported to IntAct and the data was properly re-curated, and an erratum was published (42).

As is shown in Figure 6, the incorrect interactions created by the error consistently received a low MIscore, when compared with the correctly annotated data, which has scored more highly as it has been confirmed by additional interaction evidences present in the database. Similarly, false-positive data generated by a single technique would be expected to receive a lower score than a ‘true’ interaction which has been confirmed by multiple methods. This demonstrates the value of merging data obtained by detailed literature curation with interactions evidences obtained from high-throughput protein interaction experiments and utilizing MIscore to provide a numerical assessment scoring of confidence in each interaction evidence within a dataset.

## Discussion

In this work, we present MImerge and MIscore, which provide simple scoring heuristics for molecular interactions dependent on available interaction evidence, thus providing a framework to integrate and score literature curated interaction datasets. There are multiple algorithms merging and scoring interactions (Table 1). Ten out of 27 PSICQUIC services explicitly state the use of MIscore while another 10 use a different algorithm. However, most of them are not reported in the scientific literature (to our knowledge, STRING is the only algorithm currently published (35)).

MIscore differs from other scoring methods in that it requires the minimum information needed for reporting a molecular interaction experiment to score an interaction, while other scoring algorithms depend on external data, either based on orthology detection, or ‘gold standard’ reference sets. The algorithms are customizable by the user, who can weight the interaction detection method and interaction type according to their own confidence in the different methodologies and also alter the maximum number of publications they wish to score. Default values have been supplied and used throughout in the examples.

MIscore and MImerge can help in resolving conflicting or erroneous information on molecular interactions provided by third parties. We have outlined an actual example of how the results of MImerge and MIscore were used to assess confidence levels for a high-throughput protein interaction dataset and consistently assigned low scores to an erroneous subset within it, thus demonstrating the practical relevance of the schema. Based on our experience, MImerge and MIscore can thus be used for identifying molecular interactions in interaction databases that are wrongly annotated.

With MIscore and MImerge come a set of associated tools, which together allow the user to easily access these two algorithms. The tools have been created both for bench-researchers and also for third-party services that need to integrate and measure interacting molecule pairs. While providing community agreed default settings, MIscore is customizable for specific use cases.

## Funding

This work was supported by BMBF Project 315737 (Virtual Liver Network), the Max Planck Society, NHLBI Proteomics Center Award (HHSN268201000035C), European Commission Grants PSIMEx (FP7-HEALTH-2007-223411) and European Commission Grant Affinomics (FP7-241481). Funding for open access charge: Max Planck Society.
